# *In vivo* study on the performance of therapeutic intraocular lens loaded with an antibiotic and an anti-inflammatory

**DOI:** 10.1080/07853890.2021.1896109

**Published:** 2021-09-28

**Authors:** Ana Topete, Junmei Tang, Quankui Lin, Benilde Saramago, Ana Paula Serro

**Affiliations:** a Centro de Química Estrutural, Instituto Superior Técnico, University of Lisbon, Lisbon, Portugal; b Centro de Investigação Interdisciplinar Egas Moniz (CiiEM), Egas Moniz Cooperativa de Ensino Superior, Caparica, Portugal; c School of Ophthalmology & Optometry, Eye Hospital, Wenzhou Medical University, Wenzhou, China; d Wenzhou Institute of Biomaterials and Engineering, Wenzhou, China

## Abstract

**Introduction:**

Cataract treatment usually involves surgery for substitution of the opacified eye lens by an artificial intraocular lens (IOL). In the post-operatory period, antibiotics and anti-inflammatories eye drops are prescribed to prevent endophthalmitis that may occur due to bacterial infection and results in serious complications [1,2]. This drug delivery method leads to a low bioavailability of the drugs due to the ocular clearance and absorption mechanisms. Furthermore, since it requires frequent administrations, it is uncomfortable for patients and it may lead to a low compliance. Drug-loaded intraocular lenses (IOL) have been explored as potential drug release vehicles due to their prolonged time of contact with the eye and constitute a promising alternative to eye drops [3]. The main goal of this work is to evaluate the *in vivo* performance of dual drug-loaded IOLs containing an antibiotic and an anti-inflammatory.

**Materials and methods:**

An antibiotic, moxifloxacin (MXF), and an anti-inflammatory, ketorolac (KTL) were loaded in commercial acrylic IOLs by soaking in drug solution containing the two drugs (5 mg/mL each drug) at 60 °C for 2 weeks. The effect of the drug loading on lenses properties such as the swelling capacity, optical properties (transmittance) and mechanical properties (Young’s modulus) was evaluated. After sterilisation, the drug loaded lenses were used for *in vitro* drug release tests, carried out in sink conditions (PBS, 3 mL, 36 °C, 180 rpm) and *in vivo* experiments with rabbits. A mathematical model was applied to the *in vitro* results to predict the *in vivo* concentrations. In the *in vivo* tests, the lenses were implanted into the right eye of 5 Japanese rabbits. No eye drops were administered in the post-operatory period. To evaluate ocular inflammation, slit-lamp examinations were done on the days 1, 3, 7, 14 and 21. On the day 21 the animals were anaesthetised and killed humanely with air embolism. The eyes were enucleated for histological investigation, in particular the cornea and the iris were separated, sectioned with a cryostat and stained following the haematoxylin and eosin staining.

**Results and discussion:**

It was found that the presence of drugs increases the swelling capacity of the lenses, slightly decreases the Young’s modulus and does not affect the transmittance in the range 500–700 nm. *In vitro* tests show that the lenses are able to release both drugs in a sustained way. The mathematical model indicates that the *in vivo* concentration of MXF should be higher than the minimal inhibitory concentration (MIC) of *Staphylococcus aureus* and *Staphylococcus epidermidis* (two of the most common bacteria responsible for endophthalmitis), for at least 15 days and that the concentration of KTL stays above half maximal inhibitory concentration (IC50) of cyclooxygenase 1 (and cyclooxygenase 2 (2 enzymes responsible for inflammation) for 16 days. In the *in vivo* tests, the slit-lamp examinations demonstrated that after 7 days no inflammation was present on the eyes of the rabbits. The histological evaluation proved good biocompatibility of the double loaded lenses.

**Conclusions:**

The double loaded lenses revealed to be promising devices for the post-cataract surgery prophylaxis, complying with both antibiotic and anti-inflammatory therapeutic needs.
